# Mcl-1 protects prostate cancer cells from cell death mediated by chemotherapy-induced DNA damage

**DOI:** 10.18632/oncoscience.231

**Published:** 2015-09-01

**Authors:** Teresita Reiner, Alicia de las Pozas, Ricardo Parrondo, Deanna Palenzuela, William Cayuso, Priyamvada Rai, Carlos Perez-Stable

**Affiliations:** ^1^ Geriatric Research, Education, and Clinical Center and Research Service, Bruce W. Carter Veterans Affairs Medical Center, Miami, FL, USA; ^2^ Division of Gerontology & Geriatric Medicine, Department of Medicine, University of Miami Miller School of Medicine, Miami FL, USA; ^3^ Sylvester Comprehensive Cancer Center, University of Miami Miller School of Medicine, Miami FL, USA

**Keywords:** DNA damage, apoptosis, necrosis, antimitotic, proteotoxic stress

## Abstract

The anti-apoptotic protein Mcl-1 is highly expressed in castration-resistant prostate cancer (CRPC), resulting in resistance to apoptosis and association with poor prognosis. Although predominantly localized in the cytoplasm, there is evidence that Mcl-1 exhibits nuclear localization where it is thought to protect against DNA damage-induced cell death. The role of Mcl-1 in mediating resistance to chemotherapy-induced DNA damage in prostate cancer (PCa) is not known. We show in human PCa cell lines and in TRAMP, a transgenic mouse model of PCa, that the combination of the antimitotic agent ENMD-1198 (analog of 2-methoxyestradiol) with betulinic acid (BA, increases proteotoxic stress) targets Mcl-1 by increasing its proteasomal degradation, resulting in increased γH2AX (DNA damage) and apoptotic/necrotic cell death. Knockdown of Mcl-1 in CRPC cells leads to elevated γH2AX, DNA strand breaks, and cell death after treatment with 1198 + BA- or doxorubicin. Additional knockdowns in PC3 cells suggests that cytoplasmic Mcl-1 protects against DNA damage by blocking the mitochondrial release of apoptosis-inducing factor and thereby preventing its nuclear translocation and subsequent interaction with the cyclophilin A endonuclease. Overall, our results suggest that chemotherapeutic agents that target Mcl-1 will promote cell death in response to DNA damage, particularly in CRPC.

## INTRODUCTION

Prostate cancer (PCa) is one of the leading causes of cancer-related death in men, especially when metastasis occurs. Although initially responsive to androgen deprivation therapy, PCa cells adapt to proliferate in low androgen levels leading to castration-resistant PCa (CRPC) [1, 2]. The antimitotic drug docetaxel (Doc) confers a small survival benefit for patients with CRPC; however, only 50% of these men respond to Doc therapy and they eventually develop resistance [3, 4]. Once CRPC patients fail Doc chemotherapy, only the new antimitotic cabazitaxel confers a slightly longer overall survival, although many of these patients develop increases in adverse effects [5]. Therefore, the identification of new antimitotic agents for CRPC are required in order to overcome limitations in chemoresistance and toxicity.

A promising antimitotic agent that is non-toxic to normal cells but can inhibit the growth of a variety of cancer cells is 2-methoxyestradiol (2ME2), a normal metabolite of estradiol [6]. However, 2ME2 is rapidly metabolized to an inactive form in humans, thus limiting its clinical application [7]. ENMD-1198 (denoted as 1198) is a more stable and potent derivative of 2ME2 that is well tolerated and has clinical promise [8, 9]. However, to effectively treat CRPC, antimitotics such as 1198 will likely require combinations with additional chemotherapeutic agents. For instance, we previously showed that the combination of 1198 or Doc with ABT-737, a small molecule antagonist of the anti-apoptotic proteins Bcl-2 and Bcl-xL, enhances apoptotic cell death in PCa cells [10]. Thus, to further improve overall survival of CRPC patients, a better mechanistic understanding of antimitotic-induced CRPC cell death is required to develop more effective combinatorial treatments.

The anti-proliferative activity of Doc and 1198 results from their ability to bind microtubules, disrupt mitosis, and increase apoptosis [8, 11]. The increase in apoptosis is mainly due to stabilization of mitotic cyclin B1 and a prolonged activation of cyclin-dependent kinase 1 (Cdk1) leading to enhanced phosphorylation of Bcl-2, Bcl-xL, and Mcl-1, which then antagonizes their anti-apoptotic function [12-15]. Bcl-2, Bcl-xL, and Mcl-1 are highly expressed in CRPC, resulting in resistance to apoptosis and association with poor prognosis [16]. Bcl-2, Bcl-xL, and Mcl-1 have a well known cytoplasmic function to protect cells from apoptosis by binding to Bax and Bak, pro-apoptotic members of the Bcl-2 family, and thereby blocking mitochondrial outer membrane permeabilization (MOMP), cytochrome c release, and the activation of the caspase cascade [17]. Several studies indicate that nuclear localization of Bcl-2, Bcl-xL, and Mcl-1 has a functional role in DNA damage response (DDR) and G2/M cell cycle checkpoint [18-23]. However, little is known if cytoplasmic and/or nuclear localization of Bcl-2, Bcl-xL, or Mcl-1 mediates resistance to chemotherapy-induced DNA damage and subsequent cell death in PCa cells.

In this report, we focused on Mcl-1 and its putative protective effect against chemotherapy-induced DNA damage in the context of combinatorial treatment with antimitotic drugs. We investigated the novel combination of 1198 with betulinic acid (BA), a plant-derived small molecule that increases apoptosis specifically in cancer but not in normal cells [24]. Our results showed that the 1198 + BA combination reduced total Mcl-1, increased DNA damage-associated γH2AX, and enhanced cell death in human PC3 CRPC cells and in the TRAMP model of PCa [25]. Knockdown of Mcl-1 in PC3 cells further enhanced γH2AX and DNA strand breaks induced by doxorubicin, a known DNA damaging agent. Overall, our results suggest that Mcl-1 has an important role in protecting PCa cells from cell death induced by chemotherapy-mediated DNA damage.

## RESULTS

### 1198 reduces Mcl-1 and increases apoptosis in PCa cells

To provide context for overall chemotherapeutic efficacy, we showed that 1198 was an 8-30-fold more potent inhibitor of PCa cell viability compared to 2ME2 (Supplementary Figure 1A). Since resistance to Doc is a significant clinical problem in the treatment of CRPC [4], we sought to determine if 1198 can overcome PCa cells resistant to Doc. We developed PC3 cells resistant to Doc (PC3/DR), which may be due to overexpression of multi-drug resistance (MDR) and Bcl-2 (Supplementary Figure 1B). In contrast to Doc, 1198 effectively inhibited both PC3/DR and parental PC3 cells (Supplementary Figure 1C), suggesting that 1198 may be an effective alternative to cabazitaxel in the treatment of Doc-resistant CRPC.

Given that 1198 is a known antimitotic agent that is likely to exert its action on anti-apoptotic Bcl-2 family proteins, we sought to determine its effect on Mcl-1. In LNCaP, DU145 and PC3 cells, 1 μM 1198 increased cleaved (cl)-PARP (marker of caspase activity) and the proteasome-dependent degradation of Mcl-1. In contrast, Bcl-2 and Bcl-xL levels were similar to control with the exception of increased phosphorylated isoforms (Supplementary Figure 2A, B). Interestingly, the combination of 1198 and the proteasome inhibitor MG132 increased shorter Mcl-1S isoforms in LNCaP and PC3 cells, possibly due to elevated poly-ubiquitinated (Ub) proteins and proteotoxic stress (Supplementary Figure 2B). In PC3/DR cells, 1198 but not Doc also decreased Mcl-1 and increased cl-PARP (Supplementary Figure 2C). We then showed that shRNA knockdown of Mcl-1 sensitized LNCaP and PC3 cells to 1198-mediated cell death and increased cl-PARP (Supplementary Figure 2D). These results demonstrate the importance of Mcl-1 in protecting PCa cells from 1198-mediated apoptotic cell death.

### 1198 + BA combination enhances apoptotic and necrotic cell death in PCa cells

**Figure 1:**
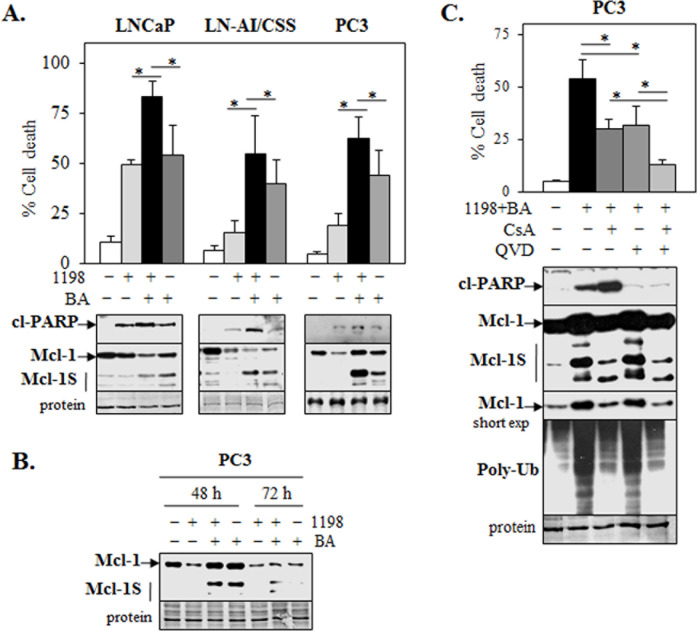
1198 + BA combination increases apoptotic and necrotic cell death in PCa cells **(A)** Trypan blue exclusion assay showed significantly greater cell death in 1198 (1 μM) + BA (10 μM) in LNCaP, LN-AI/CSS, and PC3 cells (48 h for LNCaP, LN-AI/CSS and 72 h for PC3) compared to 1198, BA, and control treated cells (*, *P<*0.04). Western blot analysis showed increased cl-PARP (24 h) in 1198 + BA compared to each alone and control in LNCaP, LN-AI/CSS, and PC3 cells. At 48 h, there was decreased Mcl-1 in 1198 + BA treated LNCaP and LN-AI/CSS but little difference in PC3. However, there was increased Mcl-1S isoforms in 1198 + BA treated PC3 cells compared to each alone and control. **(B)** Western blot analysis in PC3 cells showed decreased Mcl-1 protein at 72 h after treatment with 1198 + BA compared to 48 h. **(C)** Trypan blue exclusion assay showed that CsA (10 μM), QVD (10 μM), or CsA + QVD significantly lowered cell death in 1198 + BA treated PC3 cells at 72 h (*, *P<*0.003). Western blot analysis of PC3 cells showed that CsA but not QVD lowered the 1198 + BA (24 h) increase in Mcl-1 (short exposure [exp]), Mcl-1S isoforms, and poly-Ub accumulation. QVD but not CsA blocked the 1198 + BA increase in cl-PARP.

1198 as a single agent decreased Mcl-1 and increased cl-PARP to a greater extent in androgen-dependent LNCaP compared to PC3 CRPC cells (Supplementary Figure 2A). Therefore, it is important to identify other agents that when combined with 1198 can further reduce Mcl-1 and increase cell death in CRPC cells. We recently reported that the BA-mediated inhibition of multiple deubiquitinases (DUBs), increase in poly-Ub proteins, decrease in multiple pro-survival proteins, and induction of apoptotic cell death specifically in PCa but not in non-cancer cells could provide an effective non-toxic and clinically selective agent for chemotherapy [26]. Since we also reported that BA combined with Doc or 2ME2 stimulates apoptosis in LNCaP and non-apoptotic cell death in DU145 and PC3 cells [27], we sought to determine if the 1198 + BA combination has similar effects. Our results showed that the 1198 (1 μM) + BA (10 μM) combination significantly enhanced cell death, cl-PARP, and decreased Mcl-1 in LNCaP, LN-AI/CSS, and PC3 cells (Figure 1A). In PC3 cells, treatment with 1198 + BA initially increased Mcl-1 at 24 h when cell death was low but decreased Mcl-1 at later times (72 h) when cell death was high (Figure 1A, B). Treatment of LNCaP/shMcl-1 and PC3/shMcl-1 cells with the 1198 + BA combination resulted in increased cell death and cl-PARP, further demonstrating that Mcl-1 has a functional role in protecting cells from chemotherapy-induced cell death (Supplementary Figure 3).

In LN-AI/CSS and PC3 CRPC cells (but not as much in LNCaP), the 1198 + BA combination and BA alone increased shorter Mcl-1S isoforms previously implicated in promoting apoptosis [28, 29] (Figure 1A). In PC3 cells, the 1198 + BA-mediated increase in Mcl-1, Mcl-1S isoforms, and poly-Ub proteins was lowered by cyclosporin A (CsA), which resulted in decreased cell death, despite an increase in cl-PARP (Figure 1C). CsA is known to bind to cyclophilin D, inhibit the mitochondrial permeability transport pore (MPTP), and block certain forms of necrotic cell death [30]. Interestingly, BA is reported to increase cell death via the MPTP in a manner independent of pro-apoptotic Bax/Bak [31]. The pan-caspase inhibitor QVD also decreased cell death in 1198 + BA treated PC3 cells but had no effect on Mcl-1, Mcl-1S isoforms, or poly-Ub proteins. Combining CsA and QVD further decreased cell death mediated by the 1198 + BA combination (Figure 1C). Overall, these results in PC3 cells suggest that the 1198 + BA-mediated increase Mcl-1S isoforms may be due to a proteotoxic stress response from the accumulation of poly-Ub proteins, which may enhance caspase-dependent apoptotic and CsA-dependent necrotic cell death.

### Treatment of TRAMP mice with the 1198 + BA combination inhibits primary and metastatic PCa

**Figure 2:**
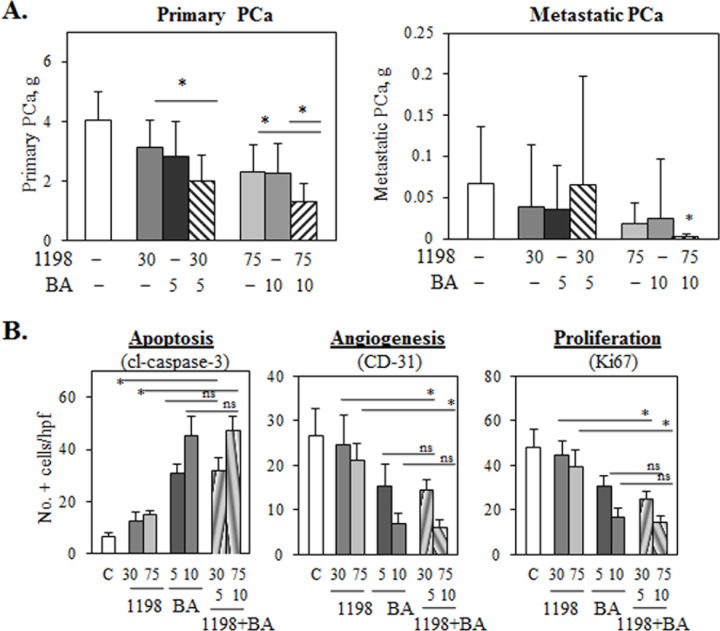
1198 + BA inhibits TRAMP primary and metastatic PCa **(A)** Weights of primary PCa were significantly less in the high dose 1198 (75 mg/kg) + BA (10 mg/kg) combination compared to 1198/75, BA/10, and vehicle control PCa (*, *P<*0.03). In the low dose 1198/30 + BA/5 combination, weights of primary PCa were significantly less compared to 1198/30 and control (*, *P<*0.02) but not to BA/5 (*P*=0.08). Weights of metastatic PCa were significantly less only between the high dose 1198/75 + BA/10 combination compared to control (*, *P<*0.009). Only 1/11 mice treated with 1198/75 + BA/10 contained visible metastatic PCa compared to 8/12 in control mice. **(B)** Number of cells immunostaining for cl-caspase-3 (apoptosis) per high powered field (hpf) were significantly increased in both low and high dose 1198 + BA combinations compared to 1198 alone and control but not to BA alone. Number of blood vessels immunostaining for CD31 (angiogenesis) or the number of cells immunostaining for Ki67 (proliferation)/hpf were significantly decreased in both low and high dose 1198 + BA combinations compared to 1198 alone and control but not to BA alone (*, *P<*0.02; ns, not significant).

To evaluate the *in vivo* therapeutic efficacy of the 1198 + BA combination, we utilized the TRAMP transgenic mouse model of PCa [25]. After first detecting palpable PCa (~0.1-0.2 g in weight), primary PCa grows rapidly and metastasizes to the pelvic lymph nodes to form visible lesions. TRAMP males with palpable PCa were treated with 1198 (30, 75 mg/kg), BA (5, 10 mg/kg), low dose 1198/30 + BA/5 combination, high dose 1198/75 + BA/10 combination, or vehicle controls for a period of two weeks (11 i.p. injections). Final weights of primary and metastatic PCa are shown in Figure 2A. Compared to 1198/75 or BA/10 alone, the high dose combination of 1198/75 + BA/10 was significantly more effective at reducing primary PCa weights by 43% (*P<*0.023). In addition, only the high dose 1198/75 + BA/10 combination was significantly more effective at reducing metastatic PCa weights by 98% (*P<*0.009) compared to vehicle controls. There were no significant differences in the final body weights between any of the treated and control mice (data not shown). Overall, these results indicate that the 1198 + BA combination is effective in the inhibition of primary and metastatic PCa in TRAMP mice.

### Decreased TRAMP PCa weights in 1198 + BA compared to BA alone does not correlate with differences in apoptosis, angiogenesis, or proliferation

We next investigated whether the significantly decreased weights of the primary PCa in the high dose 1198/75 + BA/10 combination was due to increased apoptosis and/or decreased angiogenesis or proliferation. Immunohistochemistry (IHC) of cl-caspase-3, a marker for apoptotic cells, showed a significant increase in both low and high dose 1198 + BA combinations compared to 1198 (30, 75) alone and vehicle control but not to BA (5, 10) alone (Figure 2B; Supplementary Figure 4). Similarly, IHC of CD31, a marker for blood vessels, and Ki67, a marker for proliferating cells, showed a significant decrease in both low and high dose 1198 + BA combinations compared to 1198 alone and vehicle control but not to BA alone (Figure 2B; Supplementary Figure 4). Therefore, differences in apoptosis, angiogenesis, or proliferation did not explain why primary PCa weighed significantly less in the 1198 + BA combinations compared to BA alone.

### 1198 + BA combination decreases Mcl-1 and increases DNA damage-associated γH2AX in TRAMP PCa

**Figure 3:**
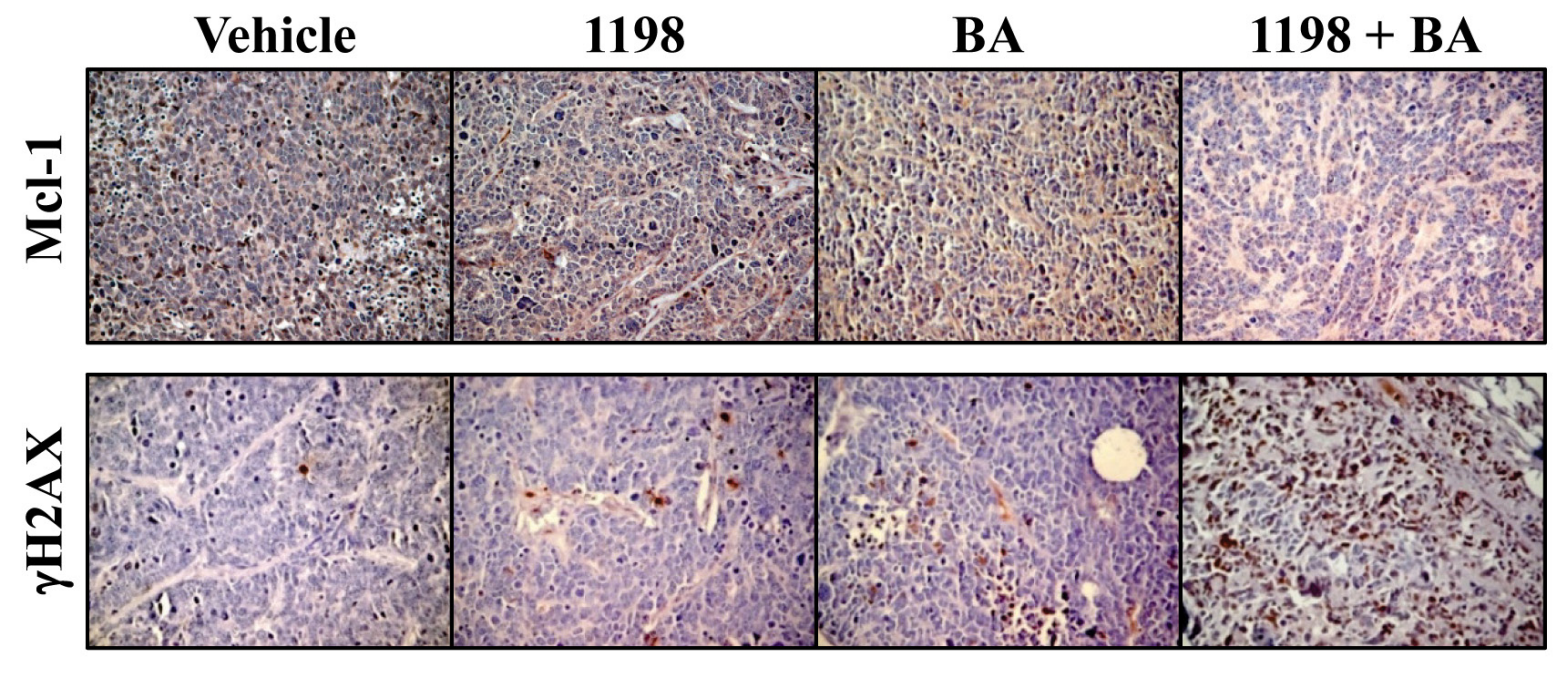
1198 + BA decreases Mcl-1 and increases DNA damage-associated γH2AX in TRAMP PCa Top panel: Representative IHC for Mcl-1 (x200, brown color) showed less protein in the high dose 1198/75 + BA/10 combination compared to 1198, BA, and vehicle control. Bottom panel: Representative IHC for γH2AX (x200, brown color) showed increased levels in the high dose 1198/75 + BA/10 combination compared to 1198, BA, and vehicle control. Similar results for Mcl-1 and γH2AX IHC were obtained from 02 additional mice from each group.

A possible reason why PCa in the high dose 1198/75 + BA/10 combination weighed less compared to 1198/75, BA/10, and vehicle controls was the lower expression of Mcl-1 (Figure 3). Since nuclear localization of Mcl-1 is reported to function in DDR [21, 22], we sought to determine in TRAMP PCa if the lower Mcl-1 in the high dose 1198/75 + BA/10 combination correlate with changes in DNA damage-associated proteins. IHC analysis of the DNA damage-associated modified histone γH2AX [32] showed that in the high dose 1198/75 + BA/10 combination there was greater γH2AX immunostaining compared to 1198, BA alone, and vehicle controls (Figure 3). These results suggest that an increase in DNA damage may be a reason why 1198/75 + BA/10 TRAMP PCa weighs less compared to BA/10 PCa, possibly by enhancing necrotic (non-apoptotic) cell death.

### Mcl-1 knockdown increases DNA damage in 1198 + BA and doxorubicin treated PC3 cells

**Figure 4:**
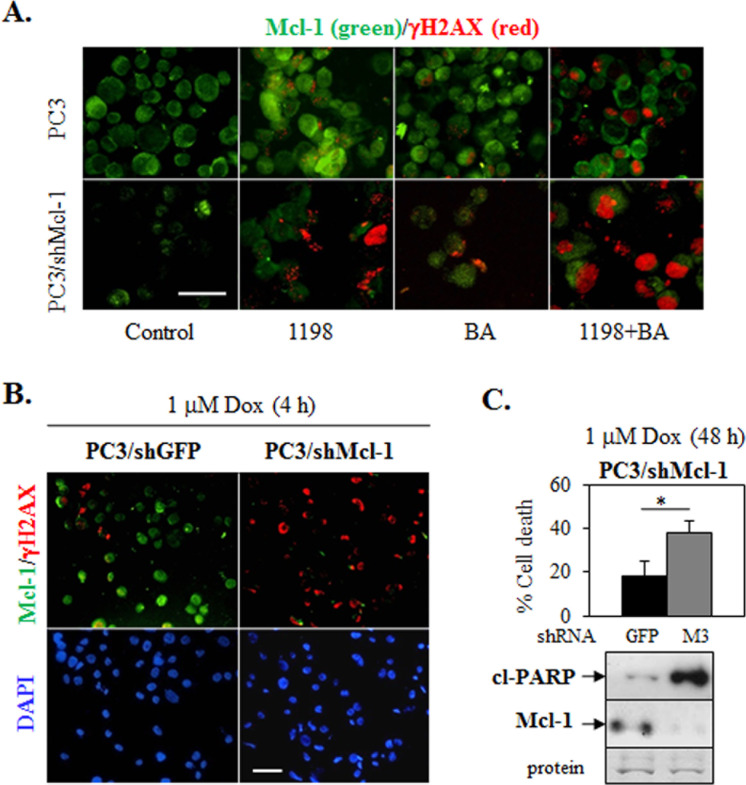
Mcl-1 knockdown in PC3 increases DNA damage-associated γH2AX immunostain after 1198 + BA or Dox treatment **(A)** DIF (top panel) showed that the 1198 + BA combination (24 h) increased immunostaining for γH2AX (red) greater than in 1198, BA, or control treated PC3 cells whereas immunostain for Mcl-1 (green) was similar (x200). In PC3/shMcl-1 cells (bottom panel), DIF immunostaining for γH2AX was further increased in the 1198 + BA combination (24 h) but less in 1198 and BA and none in control. Mcl-1 immunostaining was weak. **(B)** DIF of PC3/shMcl-1 cells treated with 1 μM Dox for 4 h showed greater immunostaining of γH2AX compared to PC3/shGFP control cells (x100). Mcl-1 immunostain was greater in PC3/shGFP compared to PC3/shMcl-1 cells. DAPI staining of nucleus is shown below DIF. **(C)** Trypan blue exclusion assay showed that 1 μM Dox (48 h) significantly increased cell death in PC3/shMcl-1 (M3) compared to PC3/shGFP control cells (*, *P*<3×10^−5^). Western blot analysis showed that 1 μM Dox (24 h) increased cl-PARP greater in PC3/shMcl-1 (low Mcl-1) compared to PC3/shGFP (high Mcl-1) control cells. White bar in (A) and (B) represents 100 μM.

We next investigated in PC3 cells if the 1198 + BA combination enhances DNA damage. Double immunoflourescence (DIF) of Mcl-1 and γH2AX showed that 1198 + BA increased γH2AX immunostain (DNA damage) greater than 1198, BA alone, and vehicle control (Figure 4A). Mcl-1 was mostly detected in the cytoplasm with variable nuclear localization; there was no clear correlation with changes in nuclear Mcl-1 and increased γH2AX after treatment. However, shRNA knockdown of Mcl-1 in PC3 cells enhanced γH2AX immunostain in the nucleus in all treatment groups (except control), especially in the 1198 + BA combination (Figure 4A). Similar results were obtained using the DNA damaging agent doxorubicin (Dox, topoisomerase II inhibitor), suggesting that Mcl-1 is protective of cell death by chemotherapy-mediated DNA damage (Figure 4B, C). There is a mixture of classical punctate and a less commonly observed diffuse pattern of γH2AX immunostaining, which may be characteristic in p53-null PC3 cells [33, 34]. Comet assay results further support a DNA damage protection role for Mcl-1 in PC3 cells treated with Dox for 4 h (Supplementary Figure 5A, B). In all cases, γH2AX immunostaining was done at time points before significant levels of cell death, indicating that DNA damage occurred before cell death (Supplementary Figure 5C).

### Possible roles of apoptosis-inducing factor and cyclophilin A in 1198 + BA-mediated DNA damage and subsequent cell death in PC3 cells

**Figure 5:**
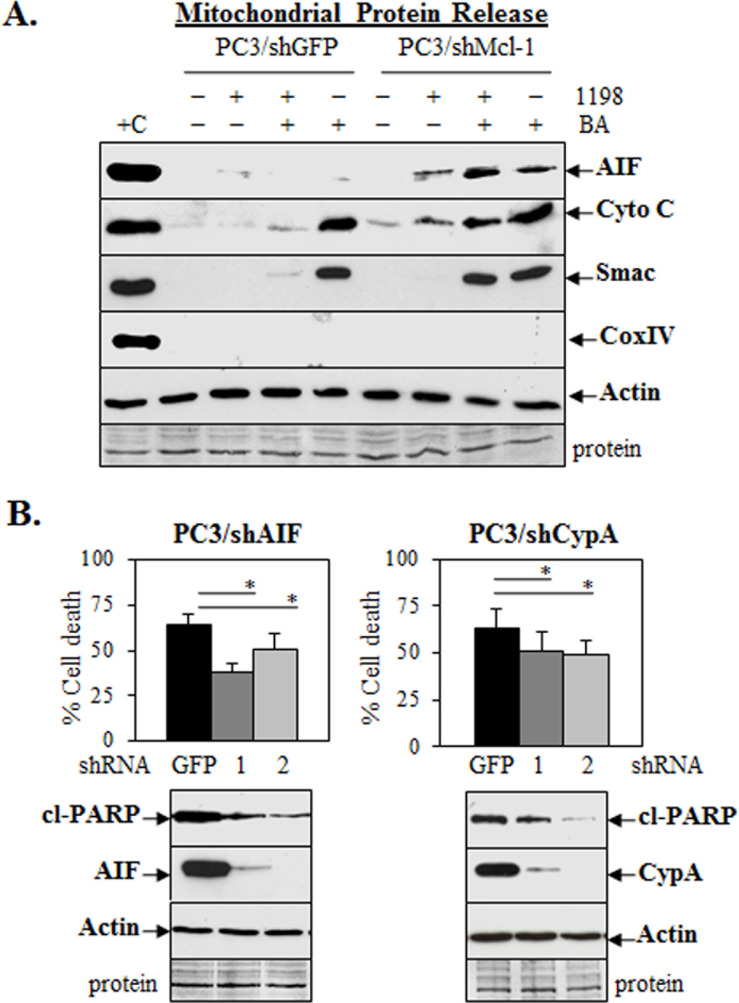
AIF or CypA knockdown in PC3 lowers cell death after 1198 + BA treatment **(A**) Mitochondrial protein release assay and western blot analysis showed increased AIF, cytochrome c, and Smac in PC3/shMcl-1 compared to PC3/shGFP cells treated with 1198 + BA for 48 h. In PC3/shMcl-1 cells, 1198 treatment increased AIF and cytochrome c but not Smac whereas BA treatment increased AIF, cytochrome c, and Smac. Cox IV protein was negative indicating no mitochondrial contamination whereas actin was the positive control. +C, lysate prepared from PC3 cells using the standard method for total proteins. **(B)** Trypan blue exclusion assay showed that 1198 + BA (72 h) significantly decreased cell death in PC3/shAIF (1, 2) and PC3/shCypA (1, 2) compared to PC3/shGFP control cells (*, *P<*0.03). Western blot analysis showed decreased cl-PARP and similar actin in PC3/shAIF (low AIF) and PC3/shCypA (low CypA) compared to PC3/shGFP cells after 1198 + BA (24 h) treatment.

Since Mcl-1 is mostly localized in the cytoplasm in PC3 cells (Figure 4A), we investigated the possibility that cytoplasmic Mcl-1 has a role in protecting PCa cells from DNA damage-induced cell death. A possible downstream cytoplasmic effector for the increase of DNA damage in PC3 cells treated with 1198 + BA is the greater release of apoptosis-inducing factor (AIF) from the mitochondria. Upon cytotoxic insult, AIF is released from the mitochondria to the cytoplasm and translocates to the nucleus where it cooperates with the endonuclease cyclophilin A (CypA) to increase DNA fragmentation [35, 36]. Our results showed that treatment of PC3/shMcl-1 cells with 1198 + BA enhanced mitochondrial release of AIF as well as cytochrome c and Smac (blocks inhibitor of apoptosis [IAP] family; [37]) greater than in PC3/shGFP cells (Figure 5A). Furthermore, treatment of LNCaP, DU145, and PC3 cells with 1198 + BA increased mitochondrial release of AIF greater than in 1198, BA, and control cells (Supplementary Figure 6).

To further investigate the relative importance of AIF and CypA in mediating 1198 + BA enhancement of cell death, we generated PC3 cells stably expressing shAIF or shCypA. Treatment of PC3/shAIF or PC3/shCypA cells with 1198 + BA resulted in decreased cell death and cl-PARP compared to PC3/shGFP control cells (Figure 5B). In PC3/shCypA cells, Dox treatment also resulted in less cell death and cl-PARP, as well as less immunostaining of γH2AX compared to PC3/shGFP cells (Supplementary Figure 7). Overall, these results suggest that a possible mechanism for how cytoplasmic Mcl-1 can protect PCa cells from DNA damage induced by chemotherapy is by blocking mitochondrial release of AIF and therefore, preventing its nuclear interaction with the CypA endonuclease.

### Increase in nuclear Mcl-1 in advanced human PCa

**Figure 6:**
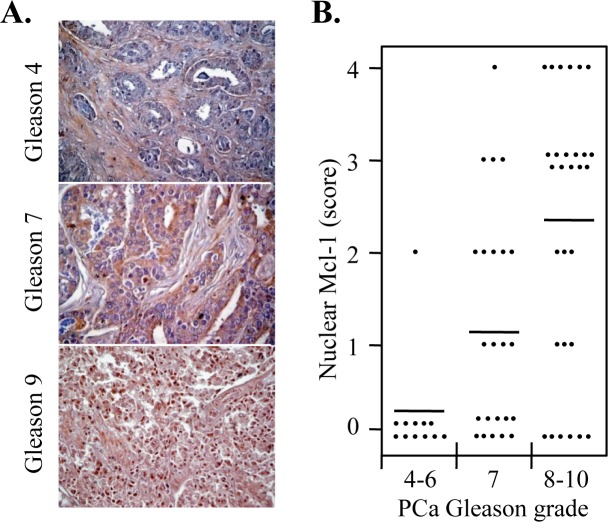
Nuclear localization of Mcl-1 is more frequent in high Gleason grade PCa **(A)** Representative IHC images (x200) of PCa tissue microarray showed increased nuclear localization of Mcl-1 (brown color) in Gleason 9 (5 + 4) compared to Gleason 4 (2 + 2) and 7 (4 + 3) PCa. **(B)** Nuclear Mcl-1 scores in the varying Gleason grades of PCa were categorized as 0 (0 to <10%), 1 (10-25%), 2 (25-50%), 3 (50-75%), or 4 (>75%). Results showed that there was very little nuclear Mcl-1 in Gleason 4-6 and an increase in Gleason 7 and 8-10 PCa tissue microarrays. Bars indicate average scores for each Gleason grade.

Previous reports demonstrate that Mcl-1 is more highly expressed in advanced PCa (Gleason grade ≥7) compared to well differentiated PCa (Gleason grade 2-4) [38, 39]. Although our *in vitro* results suggest that cytoplasmic Mcl-1 has a prominent role in protecting PC3 cells from chemotherapy-mediated DNA damage, we investigated whether there are differences in nuclear Mcl-1 localization in differing Gleason grades of PCa. Using a PCa tissue microarray, Mcl-1 was immunostained and cells positive for nuclear Mcl-1 visually scored (0 the weakest to 4 the strongest) in 64 cases categorized as Gleason grade 4-6 (n=12), 7 (n=23), and 8-10 (n=29) (representative Mcl-1 IHC pictures in Figure 6A). Our results showed that nuclear Mcl-1 was detected (score≥1) in 80% of Gleason 8-10 (23/29; average score=2.3) compared to 57% of Gleason 7 (13/23; average score=1.2), and 8.3% of Gleason 4-6 (1/12; average score=0.2) (Figure 6B; *P*<0.006). These results indicate that nuclear Mcl-1 is more common in higher Gleason (8-10) grade PCa.

## DISCUSSION

**Figure 7:**
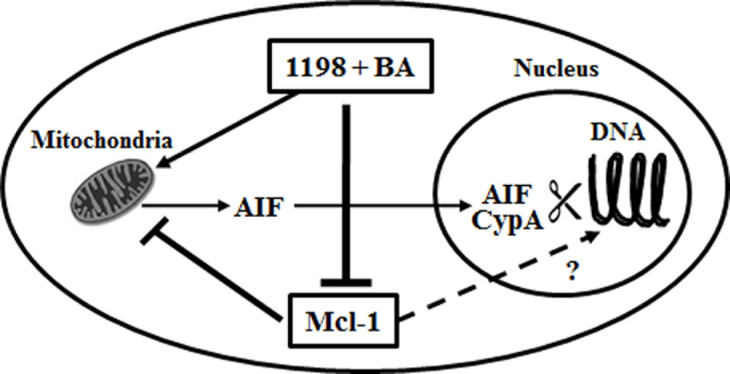
Mechanisms whereby Mcl-1 protects PCa from DNA damage inducing agents The 1198 + BA combination blocks the function of Mcl-1 by promoting its proteolytic degradation, which enhances DNA damage and multiple forms of cell death. Cytoplasmic Mcl-1 blocks MOMP and the release of AIF from the mitochondria, thus preventing translocation of AIF to the nucleus where it can associate with CypA to increase endonuclease-mediated DNA degradation. There is also a proposed role for Mcl-1 in the nucleus during DNA damage, although the mechanisms are unclear.

In addition to its well known anti-apoptotic role in the cytoplasm to prevent MOMP and the release of pro-apoptotic mitochondrial proteins, our results suggest that Mcl-1 has an important role in protecting PCa cells from DNA damage induced cell death by chemotherapeutic agents. Therefore, chemotherapy combination strategies that target Mcl-1 by 1) enhancing its proteosome-mediated destruction with antimitoic agents such as 1198 and 2) promoting proteotoxic stress and Mcl-1S pro-apoptotic isoforms with BA increases DNA damage and multiple forms of cell death. One possible mechanism is the classical cytoplasmic function of Mcl-1 (and also likely Bcl-2 and Bcl-xL) of blocking MOMP and the release of AIF from the mitochondria after treatment with chemotherapy and therefore, preventing its nuclear localization and cooperation with CypA endonuclease to degrade DNA [35, 36]. Another possible mechanism is a role for nuclear Mcl-1 during DNA damage either from treatment with chemotherapy agents or protecting high Gleason grade PCa from DNA hyper-replication or tumorigenic stress (Figure 7). Although our data does not provide a direct role for nuclear Mcl-1 in protecting PCa cells from DNA damage, there is evidence for Mcl-1 localization to sites of DNA damage, possibly as an adaptor protein [20-22].

Antimitotics such as Doc cause DNA damage and are among the most potent cytotoxic drugs in clinical use against solid tumors, including PCa [40]. However, resistance to Doc therapy often develops due to overexpression of MDR, which led to the development of cabazitaxel (does not bind MDR) as an alternative drug [5]. Our results demonstrated that unlike Doc, the antimitotic agent 1198 (more potent analog of 2ME2; [8, 9]) inhibited PC3/DR cells (high MDR expression). It is likely that 1198 is a poor substrate for MDR and therefore may be effective against CRPC resistant to Doc chemotherapy.

Our results using the combination of 1198 + BA revealed effective induction of cell death in multiple human PCa cell lines. There was a correlation between increased Mcl-1S isoforms (associated with cell death; [28, 29]) and the accumulation of poly-Ub proteins, possibly due to the ability of BA to inhibit multiple DUBs [26] (Figure 1). However, there are other Mcl-1 isoforms that may not be associated with cell death [41]; therefore, further experiments are required to determine the role of Mcl-1S isoforms in mediating cell death in PCa cells.

Mcl-1 is unique among the anti-apoptosis Bcl-2 family members because it has a very short half-life and is responsive to rapid changes in gene expression [42]. It is likely that the initial increase in total Mcl-1 at 24 h (low cell death) in PC3 cells treated with BA (or 1198 + BA) is a response to proteotoxic stress resulting from the accumulation of poly-Ub proteins. Our results also suggest that increasing MOMP with 1198 and MPTP with BA is an effective combination for enhancing DNA damage (γH2AX immunostain) and killing CRPC cells (Figures 1, 4A). The strategy of combining agents that target multiple mitochondrial cell death pathways (MOMP and MPTP) is especially important because advanced cancers such as CRPC have a weakened intrinsic pathway of apoptosis due to p53 mutations and overexpression of anti-apoptotic Bcl-2 family members [1, 2].

Our results in TRAMP mice suggested that the differences in the final primary PCa weights between the 1198+BA combination and BA alone was not due to changes in apoptosis, angiogenesis or proliferation but instead due to decreased Mcl-1, increased DNA damage (γH2AX IHC), and likely increased necrotic cell death (Figures 2, 3). Furthermore, our results in PC3 cells support a more prominent role for cytoplasmic Mcl-1 in protecting PCa cells from 1198 + BA or Dox-mediated DNA damage and cell death (Figure 4). In contrast, results in mouse embryonic fibroblasts treated with the DNA damaging agent etoposide suggests a more prominent role for nuclear Mcl-1 in supporting early DDR events such as γH2AX immunostain [21].

The ability of cytoplasmic Mcl-1 to block mitochondrial release of AIF after 1198 + BA treatment is a potential mechanism to explain its role in protecting from DNA damage-mediated cell death (Figure 5A). Interestingly, programmed necrosis induced by DNA alkylating agents results in the translocation of AIF into the nucleus where it interacts with γH2AX and helps recruit CypA to generate a DNA degradation complex [43, 44]. This form of cell death induced by chemotherapy agents may be more common in advanced cancer cells with compromised intrinsic apoptosis MOMP pathways such as in PC3 (p53 null) or TRAMP (SV40 T antigen inactivates p53 and Rb).

In conclusion, our results suggest that combining agents that target Mcl-1 will enhance cell death in response to DNA damage-inducing agents, which is especially important in advanced PCa that is inherently treatment refractory. A recent report suggesting an important role for Mcl-1 in protecting PCa cells during androgen deprivation therapy further supports chemotherapy strategies that target Mcl-1 in order to prevent or delay progression to CRPC [45].

## MATERIALS AND METHODS

### Ethics statement

All animal studies were carried out with the approval of the Institutional Animal Care and Use Committee (protocol #6996.06 MR) of the Miami Veterans Affairs Medical Center (Association for Assessment and Accreditation of Laboratory Animal Care accredited) and conducted in accordance with the NIH Guidelines for the Care and Use of Laboratory Animals.

### Reagents

1198 (3-carboxyamide-2-methoxyestra-1,3,5(10)16-tetraene) was obtained from CASI Pharmaceuticals (Rockville, MD); Doc from Sanofi-Aventis (Bridgewater, NJ); 2ME2 and polyvinyl-pyrrolidone was purchased from Sigma-Aldrich (St. Louis, MO); BA from Biovision (Milpitas, CA); CsA from Enzo Life Sciences (Farmingdale, NY); QVD from R&D Systems (Minneapolis, MN); Dox and Coomassie blue from EMD Biosciences (Temecula, CA); and trypan blue (0.4%) from Invitrogen (Carlsbad, CA). All other reagents were purchased from Sigma-Aldrich.

### Cell culture

Human PCa cell lines LNCaP, DU145, and PC3 were obtained from the American Type Culture Collection (ATCC) [33] and used within 6 months of resuscitation of original cultures. Unlike LNCaP, LN-AI cells are able to grow for long-term in RPMI 1640 with 5% charcoal-stripped fetal bovine serum (Hyclone, Logan, UT) and are referred to as LN-AI/CSS [46]. PC3 Doc resistant cells (PC3/DR) were developed by repeated exposure and recovery periods with gradual increases in Doc concentrations. LNCaP/shGFP, LNCaP/shMcl-1, PC3/shGFP, and PC3/shMcl-1 cells were developed as previously described [10]. All cells were maintained in RPMI 1640 medium (Invitrogen) with 5% fetal bovine serum (Hyclone), 100 U/ml penicillin, 100 μg/ml streptomycin, and 0.25 μg/ml amphotericin (Invitrogen). Media for viral transduced cells also contained puromycin (2 μg/ml; Invitrogen).

### Cell viability assay

The CellTiter Aqueous colorimetric method from Promega was used to determine cell viability of PCa cells in media containing 1198 (0.05-5 μM), 2ME2 (0.25-10μM), Doc (0.25-10 nM), or control (0.1% DMSO). Cell viability was normalized against the DMSO control and the data expressed as a percentage of control from three independent experiments done in triplicate.

### Lentiviral transduction

The shRNA design, lentivirus production, and infection were done as previously described [47]. The following DNA oligonucleotides (Eurofins MWG Operon, Huntsville, AL) targeting AIF and CypA were cloned into pLKO.1 lentivirus vector:

shAIF-1:CCGGCCTGGAAATAGACTCAGATTTC TCGAGAAATCTGAGTCTATTTCCAGGTTTTTG;
shAIF-2:CCGGCTGCATGCTTCTACGATATAAC TCGAGTTATATCGTAGAAGCATGCAGTTTTTG;
shCypA1:CCGGCTGACTGTGGACAACTCGAA TCTCGAGATTCGAGTTGTCCACAGTCAGTTTTTG;
shCypA2:CCGGGTTTGCAGACAAGGTCCCAA ACTCGAGTTTGGGACCTTGTCTGCAAACTTTTTG.
The control shRNA was targeted against green fluorescent protein (GFP) [10]. PC3/shAIF, PC3/shCypA, and PC3/shGFP cells were further analyzed.

### Drug treatments

PCa cells were cultured in media containing 1198 (1 μM), Doc (1 nM), BA (10 μM), 1198 + BA, Dox (1 μM), CsA (10 μM), QVD (10 μM), 1198 + BA + CsA/QVD, or DMSO (0.1%) control for varying times (24-72 h). In all the experiments, floating and trypsinized attached cells were pooled for further analysis.

### Trypan blue exclusion assay to measure total cell death

Treated and control PCa cells were harvested, resuspended in PBS, diluted 1:1 in 0.4% trypan blue, dead blue and live non-blue cells immediately counted using a hemacytometer, and the % dead blue cells determined from at least three independent experiments done in duplicate.

### Western blot analysis

Preparation of total protein lysates and western blot analysis was done as previously described [46]. The following antibodies were used: Mcl-1 (S-19), Bcl-2 (N-19), MDR (H-241), AIF (E-1), actin (C-11), and horseradish peroxidase-conjugated secondary antibody from Santa Cruz Biotechnology (Santa Cruz, CA); Bcl-xL (#610211), cytochrome c (7H8.2C12), Smac (#612245) from BD Biosciences (San Jose, CA); cleaved PARP (9541), CoxIV (#4844) from Cell Signaling Technology (Danvers, MA); and CypA (BML-SA296) from Enzo Life Sciences. After immunodetection, our preference for loading controls was for staining of total proteins transferred to the membrane with Coomassie blue because drug treatments often affect the levels of typical housekeeping proteins such as actin or tubulin.

### Mitochondrial protein release assay

Treated and control PCa cells were resuspended in a buffer containing 100 μM digitonin, 20 mM Hepes, pH 7.5, 10 mM KCl, 1.5 mM MgCl, 1 mM EGTA, 1 mM EDTA, 1 mM DTT, 250 mM sucrose, and protease inhibitors (Roche) at 50 μl/1 × 10^6^ cells. After 5 min. on ice, cells were centrifuged 5 min. and the supernatant used for western blot analysis. Digitonin is a detergent that preferentially permeabilizes plasma membrane compared to mitochondrial membrane [48].

### Treatment of TRAMP mice

TRAMP transgenic mice (Jackson Laboratories, Bar Harbor, ME) were identified by tail biopsy and PCR as previously described [26]. 1198 (50 mg/ml), obtained in the nanocrystal colloidal dispersion formulation developed by CASI Pharmaceuticals was diluted in water. BA was obtained from Ze-Qi Xu at Advanced Life Sciences and prepared as previously described [49]. Both 1198 and BA were stored at 4°C. Mice with palpable PCa were randomly divided into experimental and control groups and injected i.p. 11 times over 14 days with 1198 (30, 75 mg/kg body weight; n=10 each dose), BA (5, 10 mg/kg; n=10 each dose), low dose 1198 + BA combination (1198/30 + BA/5; n=10), high dose 1198 + BA combination (1198/75 + BA/10; n=11), and vehicle controls (n=12). On day 15, primary PCa and visible metastases to the pelvic lymph nodes were removed and their weights determined. An outer portion of primary PCa was fixed in formalin for IHC.

### Immunohistochemistry (IHC)

In TRAMP PCa, immunostaining for apoptotic (cl-caspase-3, Cell Signaling Technology) and proliferating (Ki67, NCL-Ki67p, Leica Biosystems, Buffalo Grove, IL) cells was performed using rabbit polyclonal and biotinylated goat anti-rabbit IgG secondary antibodies (Vector Laboratories, Burlingame, CA) as previously described [50]. Blood vessel density was determined by immunostaining for CD31 using a goat polyclonal antibody (M20; Santa Cruz Biotechnology) and a biotinylated rabbit anti-goat secondary antibody. Immunostaining for Mcl-1 (S-19) was performed using a 1/50 dilution of rabbit polyclonal and biotinylated goat anti-rabbit IgG secondary antibody. Immunostaining for γH2AX (clone JBW301# 05-636; EMD Bioscience) was performed using a 1/200 dilution of mouse monoclonal and the Vector Mouse on Mouse Peroxidase Kit (Vector Laboratories) following the manufacturer's instructions. The number of cleaved caspase-3, Ki67, γH2AX positive cells and CD31 positive vessels were determined for 1198, BA, 1198 + BA, and vehicle controls (n=3-5 each group), as previously described [51]. For negative controls, we used the same concentration of mouse or rabbit IgG (Santa Cruz Biotechnology) instead of specific primary antibodies, resulting in lack of immunostaining.

### Double immunofluorescence (DIF)

Adherent and non-adherent PC3 or PC3/shMcl-1 cells were harvested after a 24 h treatment with 1198, BA, 1198 + BA, or control, applied to slides by smearing, air dried, fixed in formalin for 10 min, permeabilized with 0.1% Triton X-100/PBS for 10 min, rinsed with water, and blocked with goat serum (Vector Laboratories) for 30 min. For DIF, we simultaneously immunostained with rabbit polyclonal Mcl-1 (1/150 dilution) and mouse monoclonal γH2AX (1/200 dilution) for 1 h followed by secondary antibodies Alexa Fluor 488 (green) goat anti-rabbit IgG and Alexa Fluor 594 (red) goat anti-mouse IgG (1/500 dilution; Invitrogen) for 1 h. Mounting medium with DAPI was from Vector Laboratories. Color images were acquired using a Nikon Eclipse 90i fluorescence microscope with FITC/Texas Red filters and merged using Adobe Photoshop 7. A similar DIF experiment was done with PC3/shGFP and PC3/shMcl-1 cells treated with Dox for 4 h.

### Comet assay

To measure DNA damage, we carried out single cell gel electrophoresis under alkaline conditions using the Trevigen CometAssay kit as previously described [52, 53]. PC3/shGFP and PC3/shMcl-1 cells were treated with 1 μM Dox for 4 h and gel electrophoresis carried out at 21V for 30 min at 4 °C. A minimum of 100 individual cells per sample were scored in duplicate from two independent experiments (n=4), with the DNA tail lengths being visually categorized as either none, medium, or long in blinded scoring.

### Human PCa tissue microarray and Mcl-1 IHC

Human PCa tissue microarray PR803a was purchased from US Biomax, Inc. (Rockville, MD) and utilized for immunostaining of Mcl-1 using the methods previously described [50]. The slide contains unstained paraffin sections of 64 cases of PCa categorized as Gleason grade 4-6 (n=12), 7 (n=23), and 8-10 (n=29). Visual scoring of cells positive for nuclear Mcl-1 immunostaining divided by total number of cells counted was as 0 (0 to <10%), 1 (10-25%), 2 (25-50%), 3 (50-75%), or 4 (>75%).

### Statistical analysis

Statistical differences between drug-treated and control PCa cells and between immunostaining of cl-caspase-3, CD31, Ki67 (TRAMP PCa) or nuclear Mcl-1 (human PCa) was determined by two-tailed Student's *t*-test (unequal variance) with *P*<0.05 considered significant.

## SUPPLEMENTARY MATERIAL FIGURES


