# Supplementary material for: Mcl-1 protects prostate cancer cells from cell death mediated by chemotherapy-induced DNA damage
Source: Oncoscience. 2015 Sep 1;2(8):703–15. doi: 10.18632/oncoscience.231 (PMC4580064; doi:10.18632/oncoscience.231)
Supplement: Supplementary file 1 [file oncoscience-02-703-s001.pdf]

Reiner et al, Supplementary Figures 1-7

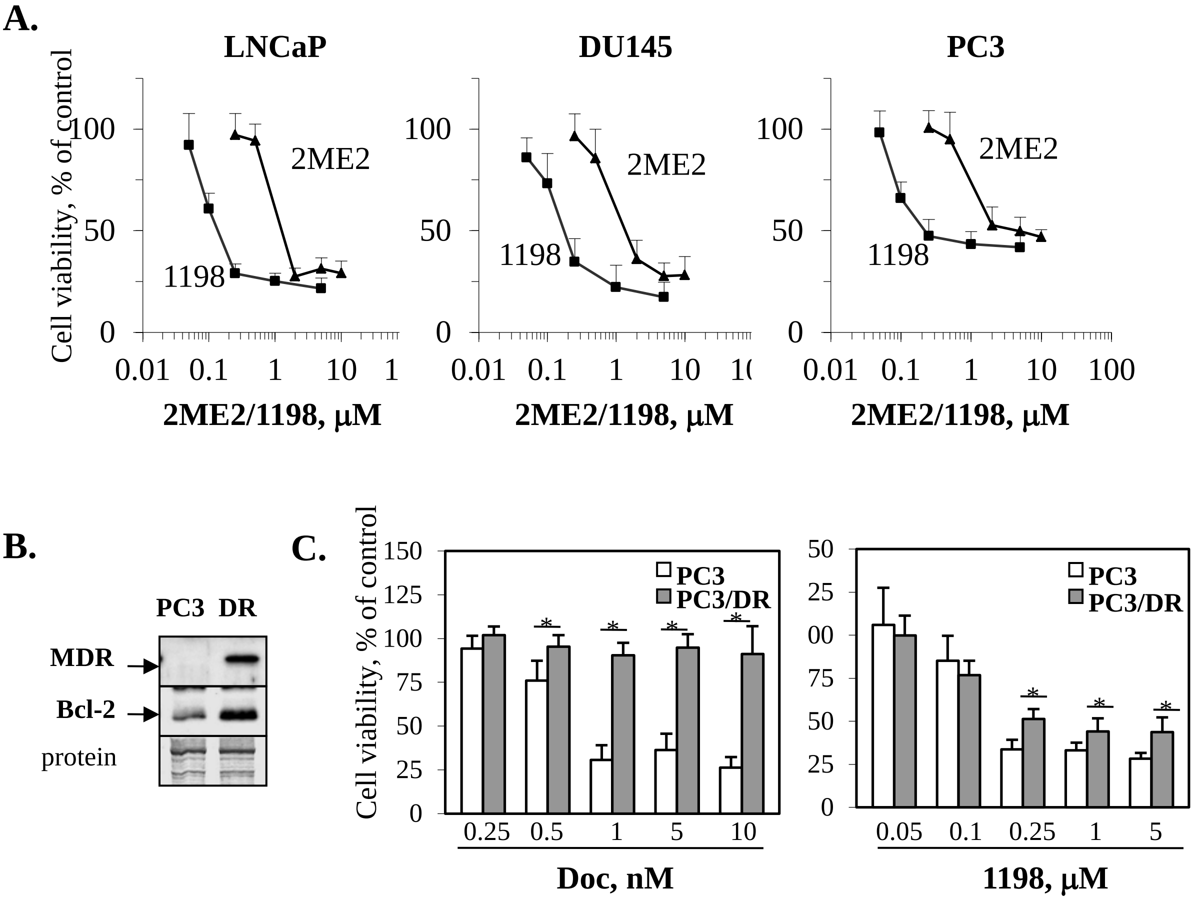

Supplementary Figure 1. 1198 is a more potent analog of 2ME2 that inhibits PC3/DR (Doc-resistant) cells. (A) Three day cell viability assay showed that increasing concentrations of 1198 (0.05-5 µM) inhibited LNCaP, DU145, and PC3 cells more effectively than 2ME2 (0.25-10 µM). **(B)** Western blot analysis showed that PC3/DR cells expressed higher levels of MDR and Bcl-2 compared to parental PC3 cells. **(C)** Cell viability assay showed that unlike parental PC3, PC3/DR cells were resistant to Doc (left panel) but not to 1198 (right panel). PC3/DR cells were slightly more resistant to 1198 (0.25, 1, and 5 µM) compared to parental PC3 cells. *, *P*<0.003.

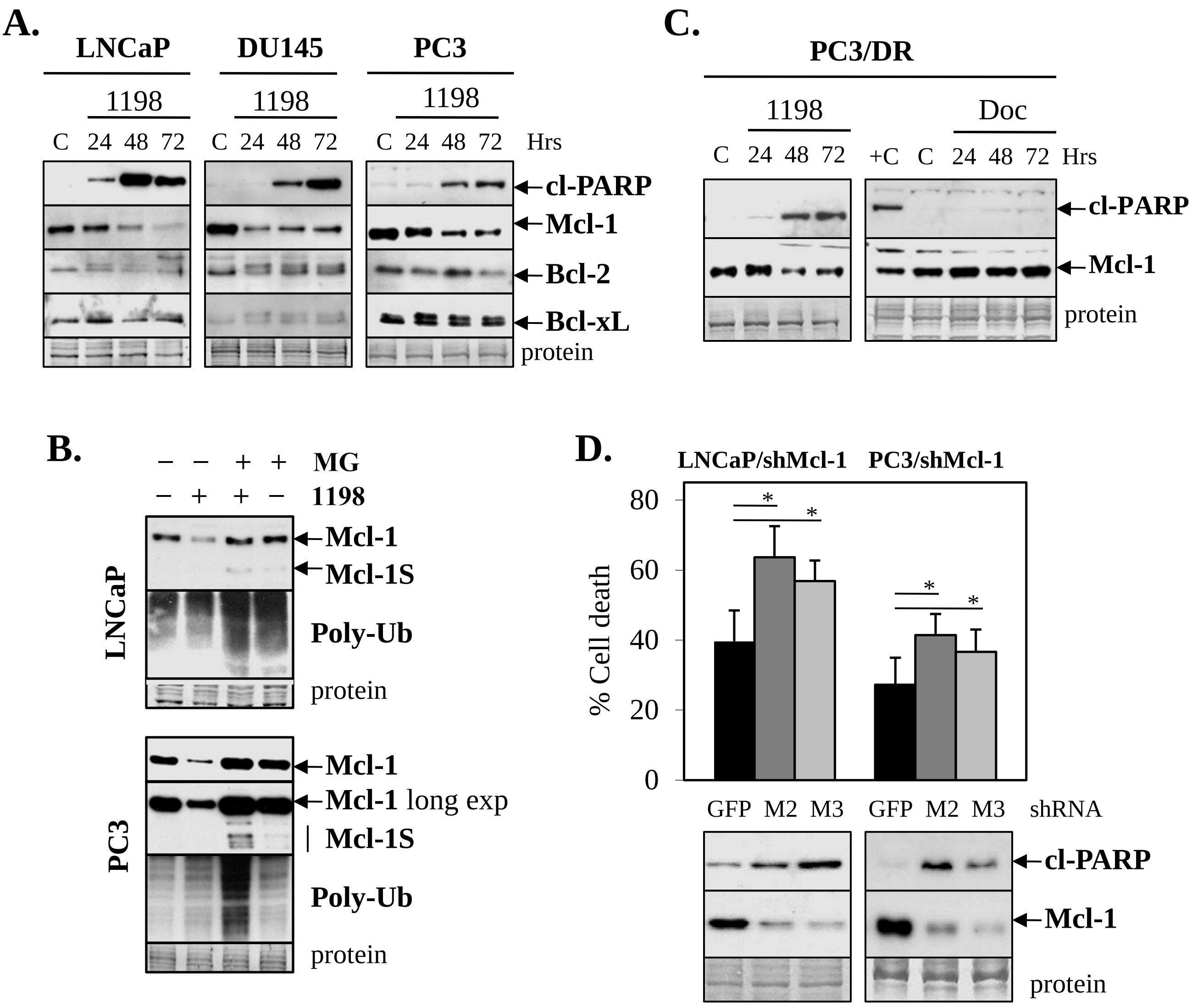

Supplementary Figure 2. **1198 reduces Mcl-1 in PCa cell lines to increase apoptosis. (A)** Western blot analysis showed that treatment of LNCaP, DU145, and PC3 cells with 1 μM 1198 reduced Mcl-1 and increased cl-PARP with time compared to control (C; 0.1% DMSO) cells. Bcl-2 and Bcl-xL levels remained similar to control except for an increase in phosphorylated isoforms (slower migrating band, as we previously determined in [10] using phospho-specific Bcl-2/Bcl-xL antibodies). After detection, Coomassie blue stain of total protein transferred to the membrane was the loading control. **(B)** Western blot analysis showed that addition of the proteasome inhibitor MG132 (MG, 1 μM) blocked the 1198-mediated degradation of Mcl-1 at 48 h in LNCaP (top) and PC3 (bottom). MG and MG + 1198 also increased poly-Ub proteins, which correlated with increased shorter Mcl-1S isoforms in LNCaP and PC3 (long exposure). **(C)** Western blot analysis showed that treatment of PC3/DR cells with 1198 but not Doc (1 nM) resulted in decreased Mcl-1 and increased cl-PARP. +C, parental PC3 treated with Doc. **(D)** Trypan blue exclusion assay showed significantly greater cell death in LNCaP/shMcl-1 (M2, M3) and PC3/shMcl-1 (M2, M3) cells treated with 1198 (48 h for LNCaP and 72 h for PC3) compared to shGFP control cells (*, *P*<0.04). Western blot analysis showed increased cl-PARP and decreased Mcl-1 in LNCaP/shMcl-1 and PC3/shMcl-1 cells treated with 1198 (16 h for LNCaP and 24 h for PC3) compared to shGFP control cells.

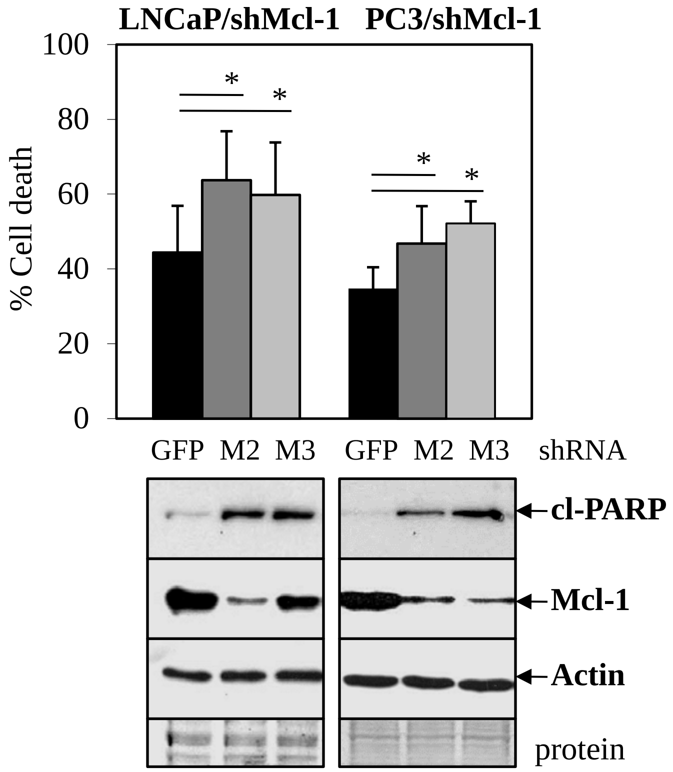

Supplementary Figure 3. Trypan blue exclusion assay showed significantly greater cell death in LNCaP/shMcl-1 (M2, M3) and PC3/shMcl-1 (M2, M3) cells treated with 1198 + BA (24 h for LNCaP and 48 h for PC3) compared to shGFP control cells (*, $P<0.04$). Western blot analysis showed increased cl-PARP, decreased Mcl-1, and similar actin in LNCaP/shMcl-1 and PC3/shMcl-1 cells treated with 1198 + BA (8 h for LNCaP and 24 h for PC3) compared to shGFP control cells.

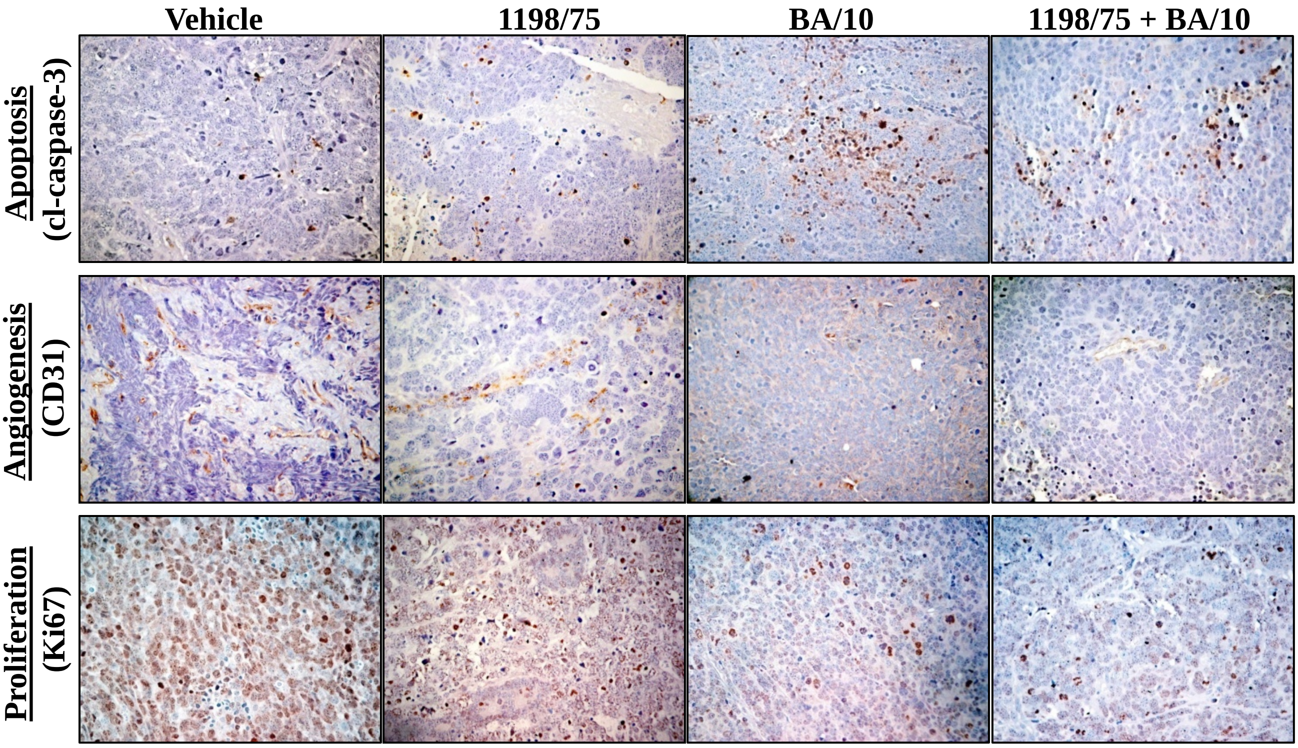

Supplementary Figure 4. Representative immunostaining of TRAMP PCa for cl-caspase-3 (apoptosis) showed increased protein in the high dose 1198/75 + BA/10 combination compared to 1198/75 and vehicle control but similar levels compared to BA/10. Representative immunostaining for CD31 (angiogenesis) and Ki67 (proliferation) showed decreased levels in the high dose 1198/75 + BA/10 combination compared to 1198/75 and vehicle control but similar levels compared to BA/10.

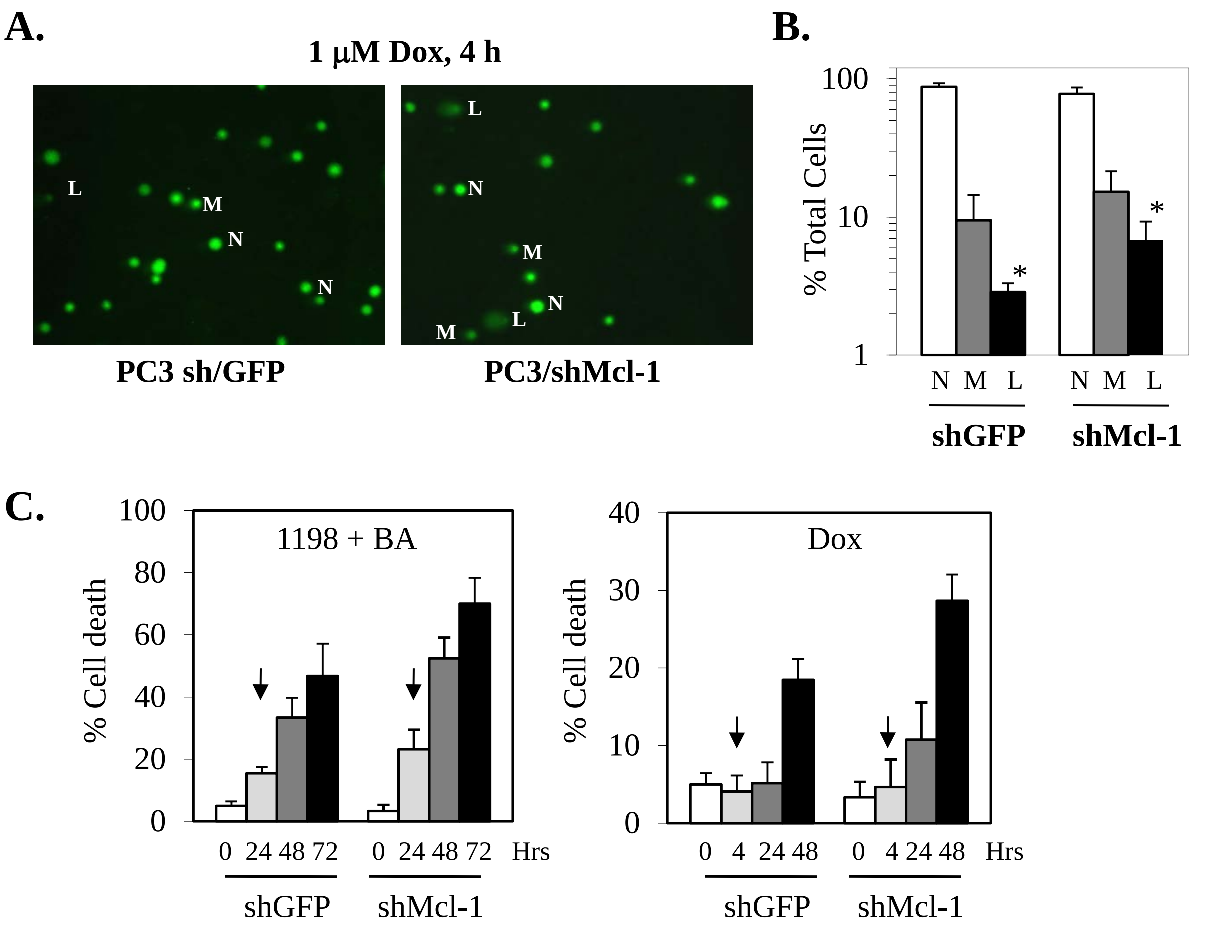

Supplementary Figure 5. **(A)** Representative images from comet assay indicating no (N), medium (M), and long (L) tails from PC3/shGFP and PC3/shMcl-1 cells treated with Dox (1 μM) for 4 h. **(B)** Quantification of tails from two independent experiments done in duplicate indicating greater long (L) tails in PC3/shMcl-1 compared to PC3/shGFP (*, $P<0.03$). Note that y-axis is logarithmic scale. **(C)** Trypan blue exclusion assay showed that 1198 + BA (left panel) and Dox (right panel) increased greater cell death in PC3/shGFP and PC3/shMcl-1 cells at time points after γH2AX immunostaining (arrows).

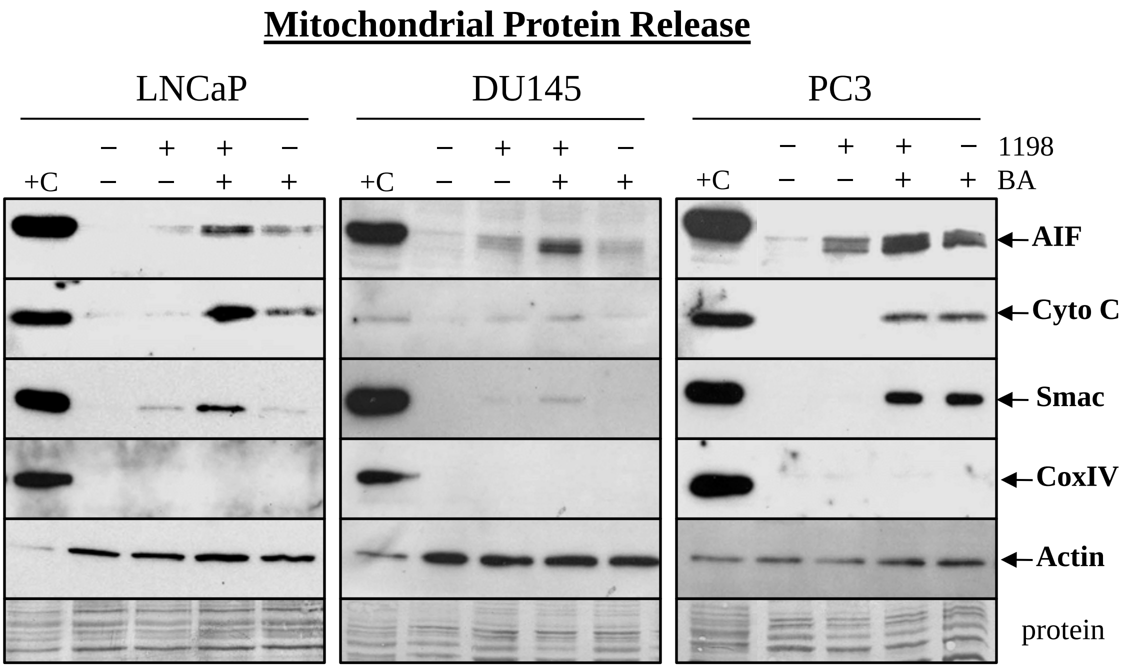

SupplementaryFigure 6. Mitochondrial protein release and western blot analysis showed increased AIF in 1198 + BA compared to 1198, BA, and control treated LNCaP (24 h), DU145 (48 h), and PC3 (72 h) cells. Cytochrome c and Smac were increased in LNCaP and DU145 but not in PC3 cells after 1198 + BA compared to BA treatment. Cox IV protein was negative indicating no mitochondrial contamination whereas actin was the positive control. +C, lysate prepared from LNCaP, DU145, and PC3 cells using the standard method for total proteins.

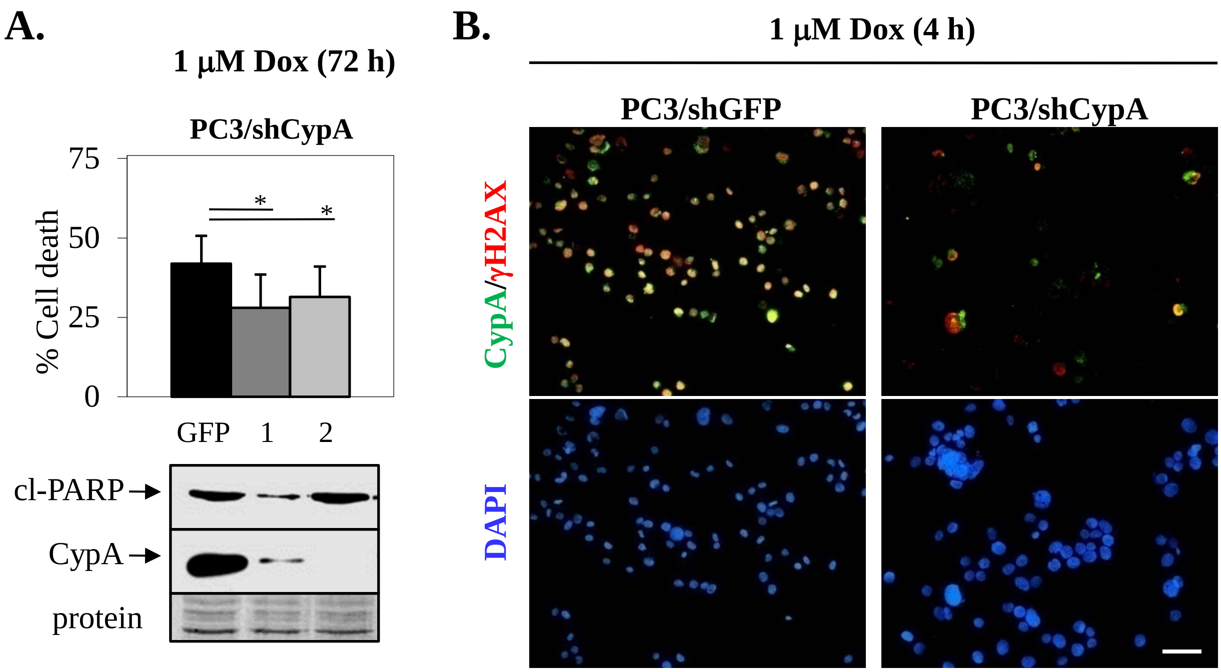

Supplementary Figure 7. **(A)** Trypan blue exclusion assay showed significantly less cell death in Dox (1 µM, 72 h) treated PC3/shCypA (1, 2) compared to PC3/shGFP cells (*, *P*<0.02). Western blot analysis showed a variable effect on cl-PARP in Dox treated (72 h) PC3/shCypA (low CypA) compared to PC3/shGFP cells (high CypA). **(B)** DIF of PC3/shCypA (clone 2) cells treated with 1 µM Dox for 4 h showed less immunostaining of γH2AX (red) compared to PC3/shGFP control cells (x100). CypA (green) immunostain was greater in PC3/shGFP compared to PC3/shCypA cells. Note that the merge of CypA and γH2AX results in numerous yellow cells, indicating co-localization in the nucleus. DAPI staining of nucleus is shown below DIF. White scale bar represents 100 µM.
